# Clinical and Demographic Factors Associated With Diabetic Retinopathy Among Young Patients With Diabetes

**DOI:** 10.1001/jamanetworkopen.2021.26126

**Published:** 2021-09-27

**Authors:** Michael L. Ferm, Daniel J. DeSalvo, Laura M. Prichett, James K. Sickler, Risa M. Wolf, Roomasa Channa

**Affiliations:** 1 Baylor College of Medicine, Texas Children’s Hospital, Houston; 2 Pediatric Endocrinology and Metabolism, Baylor College of Medicine, Texas Children’s Hospital, Houston; 3 Biostatistics, Epidemiology, and Data Management Core, Johns Hopkins University School of Medicine, Baltimore, Maryland; 4 Division of Endocrinology, Department of Pediatrics, Johns Hopkins University School of Medicine, Baltimore, Maryland; 5 Department of Ophthalmology and Visual Sciences, University of Wisconsin, Madison

## Abstract

**Importance:**

Diabetic retinopathy (DR) is a leading cause of vision loss worldwide. As the incidence of both type 1 and type 2 diabetes among youths continues to increase around the world, understanding the factors associated with the development of DR in this age group is important.

**Objective:**

To identify factors associated with DR among children, adolescents, and young adults with type 1 or type 2 diabetes in the US.

**Design, Setting, and Participants:**

This cross-sectional study pooled data from 2 large academic pediatric centers in the US (Baylor College of Medicine/Texas Children’s Hospital [BCM/TCH] Diabetes and Endocrine Care Center and Johns Hopkins University [JHU] Pediatric Diabetes Center) to form a diverse population for analysis. Data were collected prospectively at the JHU center (via point-of-care screening using fundus photography) from December 3, 2018, to November 1, 2019, and retrospectively at the BCM/TCH center (via electronic health records of patients who received point-of-care screening using retinal cameras between June 1, 2016, and May 31, 2019). A total of 1640 individuals aged 5 to 21 years with type 1 or type 2 diabetes (308 participants from the JHU center and 1332 participants from the BCM/TCH center) completed DR screening and had gradable images.

**Main Outcome and Measures:**

Prevalence of DR, as identified on fundus photography, and factors associated with DR.

**Results:**

Among 1640 participants (mean [SD] age, 15.7 [3.6] years; 867 female individuals [52.9%]), 1216 (74.1%) had type 1 diabetes, and 416 (25.4%) had type 2 diabetes. A total of 506 participants (30.9%) were Hispanic, 384 (23.4%) were non-Hispanic Black or African American, 647 (39.5%) were non-Hispanic White, and 103 (6.3%) were of other races or ethnicities (1 was American Indian or Alaska Native, 50 were Asian, 1 was Native Hawaiian or Pacific Islander, and 51 did not specify race or ethnicity, specified other race or ethnicity, or had unavailable data on race or ethnicity). Overall, 558 of 1216 patients (45.9%) with type 1 diabetes used an insulin pump, and 5 of 416 patients (1.2%) with type 2 diabetes used an insulin pump. Diabetic retinopathy was found in 57 of 1640 patients (3.5%). Patients with DR vs without DR had a greater duration of diabetes (mean [SD], 9.4 [4.4] years vs 6.6 [4.4] years; *P* < .001) and higher hemoglobin A_1c_ (HbA_1c_) levels (mean [SD], 10.3% [2.4%] vs 9.2% [2.1%]; *P* < .001). Among those with type 1 diabetes, insulin pump use was associated with a lower likelihood of DR after adjusting for race and ethnicity, insurance status, diabetes duration, and HbA_1c_ level (odds ratio [OR], 0.43; 95% CI, 0.20-0.93; *P* = .03). The likelihood of having DR was 2.1 times higher among Black or African American participants compared with White participants (OR, 2.12; 95% CI, 1.12-4.01; *P* = .02); this difference was no longer significant after adjusting for duration of diabetes, insurance status, insulin pump use (among patients with type 1 diabetes only), and mean HbA_1c_ level (type 1 diabetes: OR, 1.79; 95% CI, 0.83-3.89; *P* = .14; type 2 diabetes: OR, 1.08; 95% CI, 0.30-3.85; *P* = .91).

**Conclusions and Relevance:**

This study found that although the duration of diabetes and suboptimal glycemic control have long been associated with DR, insulin pump use (among those with type 1 diabetes) was independently associated with a lower likelihood of DR, which is likely owing to decreased glycemic variability and increased time in range (ie, the percentage of time blood glucose levels remain within the 70-180 mg/dL range). Black or African American race was found to be associated with DR in the univariable analysis but not in the multivariable analysis, which may represent disparities in access to diabetes technologies and care.

## Key Points

**Question:**

What factors are associated with diabetic retinopathy (DR) among children, adolescents, and young adults with diabetes in the US?

**Findings:**

In this cross-sectional study of 1640 young patients with type 1 or type 2 diabetes, DR was present in 3.5% of patients at the time of screening; risk factors included duration of diabetes and higher mean hemoglobin A_1c_ level. The use of insulin pumps was associated with a lower likelihood of DR; racial and ethnic differences in DR prevalence were not significant after adjusting for insurance status, hemoglobin A_1c_ level, insulin pump use (among those with type 1 diabetes), and duration of disease.

**Meaning:**

In this study, young patients with type 1 diabetes who used insulin pumps were less likely to have DR independent of other risk factors.

## Teaser

This cross-sectional study pools data from 2 large pediatric academic centers to examine factors associated with diabetic retinopathy among children, adolescents, and young adults with type 1 or type 2 diabetes in the US.

## Introduction

The incidence of both type 1 and type 2 diabetes among youths continues to increase around the world.^1,2,3^ Given this trend, understanding the factors associated with sequelae is increasingly important. Diabetic retinopathy (DR) is one such complication that is consistently cited as a leading cause of vision loss worldwide.^4,5^ Results of the Diabetes Control and Complications Trial suggested that intensive insulin therapy was associated with reductions of up to 76% in the incidence of microvascular complications among adults with diabetes.^6^ In the resulting era of intensive insulin therapy, the prevalence of DR has decreased alongside a reduction in hemoglobin A_1c_ (HbA_1c_) levels among people with diabetes,^5,7^ which is encouraging and highlights the need for interval studies as treatment modalities continue to improve.

Studies have examined the prevalence of both pediatric and adult DR in multiple countries,^1,8,9,10,11,12,13,14,15^ but within the US, the largest studies have focused on adults with diabetes.^16^ Although large pediatric cohorts from countries with national diabetes registries have been examined,^17^ US children, adolescents, and young adults are an increasing and diverse group who are not well represented by these studies. In addition, diabetes technologies are expanding rapidly.^18,19^ Monitoring the impact of new technologies for diabetes control and outcomes is important to practicing evidence-based medicine.

Screening for DR is recommended by multiple professional organizations.^20,21,22^ Based on the most recent American Diabetes Association guidelines, screening for DR using a dilated eye examination is recommended for young patients who have had type 1 diabetes for 3 to 5 years, provided that they are 11 years or older or have started puberty, whichever comes first. Among young patients with type 2 diabetes, screening is recommended at diagnosis and annually thereafter.^20^ Although a dilated and comprehensive eye examination by an ophthalmologist remains the standard of care for retinal screening, fundus photography with or without artificial intelligence–based techniques for point-of-care detection of DR serves as an accurate screening tool for DR, is feasible for use among young patients with diabetes, increases adherence to recommended screening, and is cost-effective.^23,24,25,26,27,28^

Our study sought to estimate the burden of DR among children, adolescents, and young adults in the US at the time of point-of-care DR screening and to identify factors associated with DR by combining data from 2 large pediatric hospitals that have robust DR screening programs. Combining data from these 2 sites allowed us to report findings from analyses that, to our knowledge, include one of the largest and most racially diverse groups of young patients with type 1 and type 2 diabetes in the US.

## Methods

This cross-sectional study was approved by the institutional review boards of both Baylor College of Medicine (BCM) and Johns Hopkins University (JHU) School of Medicine in adherence to the Declaration of Helsinki.^29^ All participants and parents provided written informed consent/assent. This study followed the Strengthening the Reporting of Observational Studies in Epidemiology (STROBE) reporting guideline for cross-sectional studies.^30^

The JHU Pediatric Diabetes Center is a multidisciplinary comprehensive diabetes program located in the Baltimore, Maryland, metropolitan area. Methods for data collection have been described previously.^26^ In brief, point-of-care DR screening was performed during routine care visits between December 3, 2018, and November 1, 2019, as part of a prospective study of patients aged 5 to 21 years with type 1 or type 2 diabetes. Two color images per eye (1 macula-centered and 1 disc-centered) were obtained using a nonmydriatic fundus camera (TRC NW400; Topcon Corp) and graded independently by 2 retina specialists (one of whom was R.C.) for DR presence and severity, with discrepant interpretations adjudicated by a third retina specialist.

The BCM/Texas Children’s Hospital (TCH) Diabetes and Endocrine Care Center comprises 6 clinics in the Houston metropolitan area. Retrospective data from patients who received point-of-care DR screening via retinal cameras between June 1, 2016, and May 31, 2019, were extracted from the electronic health record (Epic software; Epic Systems Corp). For patients with multiple fundus images within the study period, only the most recent images were used. Patient data and characteristics used in our analyses were from the day of fundus photography. One retinal image of each eye capturing the macula and disc was acquired in the pediatrics clinics using a nonmydriatic auto fundus camera (AFC-230; NIDEK); images were reviewed remotely and initially classified as normal, abnormal, or other. Images classified in the other category were excluded from further analysis because these images were deemed ungradable by the reviewers. All abnormal images were again reviewed by one of the coauthors (R.C.) to determine DR grade.

Demographic and clinical data, including patient age, patient-reported sex, patient-reported race and ethnicity, insurance status (private/commercial vs public/Medicaid), duration of diabetes based on clinician-reported date of diagnosis, type of diabetes, clinical measurements,^31^ medical history, receipt of medications, laboratory results, and fundus examination results were collected from the electronic health record via standardized data extraction of explicitly defined variables by one of the authors (J.K.S.). For analytic purposes, race and ethnicity were grouped by the authors into Hispanic, non-Hispanic Black or African American, non-Hispanic White, and other race or ethnicity (including patients who were American Indian or Alaska Native, Asian, Native Hawaiian or Pacific Islander, and those who did not specify race or ethnicity, specified other race or ethnicity, or had unavailable data on race or ethnicity); patients included in the other race or ethnicity group were analyzed together because of the small number of patients in each racial and/or ethnic category. In cases of incomplete data after extraction, manual records review was performed by another author (M.L.F.) who was not blinded to the study hypothesis.

### Statistical Analysis

Differences between participants with vs without DR were evaluated using χ^2^ tests for categorical variables and *t* tests for continuous variables. All hypothesis tests were 2-sided. Univariable logistic regression modeling was also used to further explore these differences and to identify variables for inclusion in multivariable models. Multivariable logistic regression modeling was used to identify the association of variables with the likelihood of developing DR, holding other factors in the model constant. A stratified multivariable logistic regression analysis was used to examine the role of diabetes type in the associations identified in the full multivariable models. A cutoff of 2-tailed *P* < .05 was used to determine statistical significance for all models. In the case of 2 or more colinear variables, only 1 variable was included in the multivariable regression analysis. Data were reported as frequencies with percentages, means with SDs, or odds ratios (ORs) with 95% CIs. All analyses were performed using Stata software, version 15.1 (StataCorp LLC).

## Results

At the JHU clinics, 327 patients and families were approached to participate in imaging, 310 provided informed consent to receive imaging, and 308 received imaging of sufficient quality for grading. At the BCM/TCH clinics, 5672 patients were evaluated during the study period, and 2828 met the 2018 American Diabetes Association guidelines for eye screening^32^; 1398 patients completed point-of-care fundus imaging, and 1332 had gradable images.

The total study population therefore included 1640 patients (mean [SD] age, 15.7 [3.6] years; median age, 16 years [interquartile range, 14-18 years]; 867 female participants [52.9%] and 773 male participants [47.1%]) who completed point-of-care DR screening at 1 of the 2 institutions. In total, 1216 individuals (74.1%) had type 1 diabetes, and 416 (25.4%) had type 2 diabetes. The mean (SD) age at diabetes diagnosis was 9.1 (4.2) years, and the mean (SD) duration of diabetes at the time of screening was 6.7 (4.4) years. A total of 506 participants (30.9%) were Hispanic, 384 (23.4%) were non-Hispanic Black or African American, 647 (39.5%) were non-Hispanic White, and 103 (6.3%) were of other races or ethnicities (1 was American Indian or Alaska Native, 50 were Asian, 1 was Native Hawaiian or Pacific Islander, and 51 did not specify race or ethnicity, specified other race or ethnicity, or had unavailable data on race or ethnicity) (Table 1).

**Table 1.** Participant Characteristics

Characteristic	No./total No. (%)			*P* value^a^
	All participants	Participants without DR	Participants with DR	
Total participants, No.	1640	1583	57	NA
Age at screening, mean (SD), y	15.7 (3.6)	15.7 (3.5)	18.2 (2.6)	<.001
Age at diabetes diagnosis, mean (SD), y	9.1 (4.2)	9.2 (4.2)	8.9 (4.2)	.68
Duration of diabetes, mean (SD), y	6.7 (4.4)	6.6 (4.4)	9.4 (4.4)	<.001
Sex				
Female	867/1640 (52.9)	841/1583 (53.1)	26/57 (45.6)	.26
Male	773/1640 (47.1)	742/1583 (46.9)	31/57 (54.4)	
Race and ethnicity				
Hispanic	506/1640 (30.9)	489/1583 (30.9)	17/57 (29.8)	.02
Non-Hispanic Black or African American	384/1640 (23.4)	362/1583 (22.9)	22/57 (38.6)	
Non-Hispanic White	647/1640 (39.5)	629/1583 (39.7)	18/57 (31.6)	
Other^b^	103/1640 (6.3)	103/1583 (6.5)	0	
Insurance				
Private/commercial	867/1572 (55.2)	841/1517 (55.4)	26/55 (47.3)	.23
Public/Medicaid	705/1572 (44.8)	676/1517 (44.6)	29/55 (52.7)	
BMI percentile, mean (SD)^c^	77.58 (25.06)	77.64 (25.04)	75.59 (26.16)	.63
Type of diabetes				
1	1216/1640 (74.1)	1173/1583 (74.1)	43/57 (75.4)	.35
2	416/1640 (25.4)	403/1583 (25.5)	13/57 (22.8)	
Unknown	8/1640 (0.5)	7/1583 (0.4)	1/57 (1.8)	
Use of continuous glucose monitor	433/1640 (26.4)	419/1583 (26.5)	14/57 (24.6)	.74
Use of insulin pump	564/1640 (34.4)	554/1583 (35.0)	10/57 (17.5)	.006
HbA_1c_ level				
Mean (SD), %	9.3 (2.1)	9.2 (2.1)	10.3 (2.4)	<.001
Last measurement, mean (SD), %	9.3 (2.4)	9.3 (2.4)	10.2 (2.5)	.006
Ever >8%	1329/1640 (81.0)	1278/1583 (80.7)	51/57 (89.5)	.10
Never >8%	311/1640 (19.0)	305/1583 (19.3)	6/57 (10.5)	
Endocrine appointments within past 12 mo, mean (SD), No.^d^	2.0 (1.5)	2.1 (1.5)	1.3 (1.4)	.002
Diabetic ketoacidosis admissions within past 12 mo, mean (SD), No.^d^	0.05 (0.30)	0.05 (0.30)	0.09 (0.30)	.37

Abbreviations: BMI, body mass index; DR, diabetic retinopathy; HbA_1c_, hemoglobin A_1c_; NA, not applicable.

^a^ *P* values were derived from χ^2^ test for categorical variables and *t* test for continuous variables.

^b^ Includes patients who were American Indian or Alaska Native (n = 1), Asian (n = 50), Native Hawaiian or Pacific Islander (n = 1), and did not specify race or ethnicity, specified other race or ethnicity, or had unavailable data on race or ethnicity (n = 51).

^c^ The BMI percentiles were obtained by plotting values on the Centers for Disease Control and Prevention’s BMI-for-Age charts for boys and girls.^31^

^d^ Data only available for the 1332 patients at Baylor College of Medicine/Texas Children’s Hospital.

Overall, DR was present in 57 of 1640 participants (3.5%), and the prevalence did not vary between those with type 1 vs type 2 diabetes (43 of 1216 patients [3.5%] vs 13 of 416 patients [3.1%], respectively; *P* = .35) or between institutions (13 of 308 patients [4.2%] at JHU vs 44 of 1332 patients [3.3%] at BCM/TCH; *P* = .19). Among the 57 patients with DR, 11 individuals (19.3%) had mild nonproliferative DR (non-PDR), 45 (78.9%) had moderate non-PDR, and 1 (1.8%) had PDR. Participants with DR vs without DR were older (mean [SD] age, 18.2 [2.6] years vs 15.7 [3.5] years, respectively; *P* < .001), had a longer diabetes duration (mean [SD], 9.4 [4.4] years vs 6.6 [4.4] years; *P* < .001), were less likely to use an insulin pump (10 of 57 patients [17.5%] vs 554 of 1583 patients [35.0%]; *P* = .006), and had higher mean HbA_1c_ levels (mean [SD], 10.3% [2.4%] vs 9.2% [2.1%]; *P* < .001) (Table 1).

A total of 558 of 1216 patients (45.9%) with type 1 diabetes used an insulin pump, and only 5 of 416 patients (1.2%) with type 2 diabetes used an insulin pump. Among patients with type 1 diabetes, those who used an insulin pump vs those who did not were more likely to be White (361 of 558 patients [64.7%] vs 250 of 658 patients [38.0%], respectively; *P* < .001), with pump use significantly lower among Black or African American patients (66 of 244 participants [27.0%]) compared with White patients (361 of 611 participants [59.1%]; *P* < .001). Those who used a pump vs those who did not were also more likely to have private/commercial insurance (404 of 550 patients [73.5%] vs 351 of 628 [55.9%]; *P* < .001), use a continuous glucose monitor (308 of 558 patients [55.2%] vs 116 of 658 [17.6%]; *P* < .001), have a lower mean HbA_1c_ level (mean [SD], 8.7% [1.5%] vs 10.0% [2.1%]; *P* < .001), have fewer hospital admissions for diabetic ketoacidosis (mean [SD], 0.04 [0.22] vs 0.09 [0.37] admission [1 admission per 25 pump users per year vs 1 admission per 11 nonpump users per year]; *P* = .01), and attend more endocrine appointments within the past 12 months (mean [SD], 2.5 [1.4] visits vs 1.9 [1.5] visits; *P* < .001) (Table 2).

**Table 2.** Characteristics of Participants With Type 1 Diabetes by Insulin Pump Use

Characteristic	Use of insulin pump, No./total No. (%)		*P* value^a^
	No	Yes	
Total participants, No.	658	558	NA
Age at screening, mean (SD), y	15.9 (3.7)	15.1 (3.9)	<.001
Age at diabetes diagnosis, mean (SD), y	8.3 (3.9)	7.1 (3.6)	<.001
Duration of diabetes, mean (SD), y	7.7 (4.4)	8.1 (4.0)	.14
Sex			
Female	316/658 (48.0)	299/558 (53.6)	.05
Male	342/658 (52.0)	259/558 (46.4)	
Race and ethnicity			
Hispanic	187/658 (28.4)	94/558 (16.8)	<.001
Non-Hispanic Black or African American	178/658 (27.1)	66/558 (11.8)	
Non-Hispanic White	250/658 (38.0)	361/558 (64.7)	
Other^b^	43/658 (6.5)	37/558 (6.6)	
Insurance			
Private/commercial	351/628 (55.9)	404/550 (73.5)	<.001
Public/Medicaid	277/628 (44.1)	146/550 (26.5)	
BMI percentile, mean (SD)^c^	72.2 (25.9)	70.5 (25.1)	.27
Diabetic retinopathy diagnosis	33/658 (5.0)	10/558 (1.8)	.002
Use of continuous glucose monitor	116/658 (17.6)	308/558 (55.2)	<.001
HbA_1c_, level			
Mean (SD), %	10.0 (2.1)	8.7 (1.5)	<.001
Last measurement, mean (SD), %	10.0 (2.3)	8.7 (1.6)	<.001
Ever >8%	591/658 (89.8)	438/558 (78.5)	<.001
Endocrine appointments within past 12 mo, mean (SD), No.^d^	1.9 (1.5)	2.5 (1.4)	<.001
Diabetic ketoacidosis admissions within past 12 mo, mean (SD), No.^d^	0.09 (0.37)	0.04 (0.22)	.01

Abbreviations: BMI, body mass index; HbA_1c_, hemoglobin A_1c_; NA, not applicable.

^a^ *P* values were derived from χ^2^ test for categorical variables and *t* test for continuous variables.

^b^ Includes patients who were Asian (n = 50) and those who did not specify race or ethnicity, specified other race or ethnicity, or for whom data on race or ethnicity were not available (n = 51).

^c^ The BMI percentiles were obtained by plotting values on the Centers for Disease Control and Prevention’s BMI-for-Age charts for boys and girls.^31^

^d^ Data only available for the 1332 patients at Baylor College of Medicine/Texas Children’s Hospital.

The univariable analysis revealed that DR was significantly associated with duration of diabetes (OR, 1.14; 95% CI, 1.08-1.21; *P* < .001), Black or African American race (OR, 2.12; 95% CI, 1.12-4.01; *P* = .02), and mean HbA_1c_ level (OR, 1.24; 95% CI, 1.10-1.39; *P* < .001) (Table 3). Patients who used insulin pumps (OR, 0.40; 95% CI, 0.20-0.79; *P* = .008) or had more endocrine appointments within the past 12 months (OR, 0.69; 95% CI, 0.54-0.87; *P* = .002) were less likely to have DR than those who did not. No significant differences were noted between White and Hispanic participants (OR, 1.22; 95% CI, 0.62-2.38; *P* = .57).

**Table 3.** Univariable Logistic Regression Analysis With Diabetic Retinopathy as Outcome

Variable	Odds ratio (95% CI)	SE	*z* Score	*P* value
Age at screening	1.26 (1.16-1.37)	0.05	5.33	<.001
Age at diabetes diagnosis	0.99 (0.93-1.05)	0.03	−0.41	.68
Duration of diabetes	1.14 (1.08-1.21)	0.03	4.64	<.001
Male sex	1.35 (0.80-2.30)	0.37	1.11	.27
Race and ethnicity				
Black or African American	2.12 (1.12-4.01)	0.69	2.32	.02
Hispanic	1.22 (0.62-2.38)	0.42	0.57	.57
Other^a^	1 (NA)	NA	NA	NA
White	1 [Reference]	NA	NA	NA
Insurance				
Private/commercial	1 [Reference]	NA	NA	NA
Public/Medicaid	1.39 (0.81-2.38)	0.38	1.19	.23
BMI percentile	1.00 (0.98-1.01)	0.01	−0.48	.63
Type of diabetes				
1	1 [Reference]	NA	NA	NA
2	0.88 (0.47-1.65)	0.28	−0.40	.69
Unknown	3.90 (0.47-32.38)	4.21	1.26	.21
Use of continuous glucose monitor	0.90 (0.49-1.66)	0.28	−0.33	.74
Use of insulin pump	0.40 (0.20-0.79)	0.14	−2.64	.008
HbA_1c_ level				
Mean	1.24 (1.10-1.39)	0.07	3.64	<.001
Last measurement	1.16 (1.04-1.28)	0.06	2.72	.006
Ever >8%	2.03 (0.86-4.77)	0.89	1.62	.11
Endocrine appointments within past 12 mo^b^	0.69 (0.54-0.87)	0.08	−3.14	.002
Diabetic ketoacidosis admissions within past 12 mo^b^	1.45 (0.64-3.27)	0.60	0.88	.38

Abbreviations: BMI, body mass index; HbA_1c_, hemoglobin A_1c_; NA, not applicable.

^a^ The other race and ethnicity category includes patients who were American Indian or Alaska Native (n = 1), Asian (n = 50), Native Hawaiian or Pacific Islander (n = 1), and did not specify race or ethnicity, specified other race or ethnicity, or for whom data on race or ethnicity were not available (n = 51).

^b^ Data only available for the 1332 patients at Baylor College of Medicine/Texas Children’s Hospital.

Multivariable regression models stratified by type of diabetes revealed that insulin pump use was associated with a lower likelihood of DR (OR, 0.43; 95% CI, 0.20-0.93; *P* = .03) among patients with type 1 diabetes after controlling for duration of diabetes, insurance status, race and ethnicity, and mean HbA_1c_ level (Table 4). The number of patients with type 2 diabetes who were receiving insulin pump therapy was too small to allow a multivariable analysis that included pump use. The duration of diabetes was independently associated with DR among patients with type 1 diabetes (OR, 1.18; 95% CI, 1.10-1.26; *P* < .001) and type 2 diabetes (OR, 1.21; 95% CI, 1.02-1.45; *P* = .03). After adjusting for duration of diabetes, mean HbA_1c_ level, insurance status, and insulin pump use in the multivariable regression analysis, Black or African American race was no longer associated with an increased risk of DR among patients with type 1 diabetes (OR, 1.79; 95% CI, 0.83-3.89; *P* = .14) or type 2 diabetes (OR, 1.08; 95% CI, 0.30-3.85; *P* = .91). Although insurance data were incomplete for some patients, a sensitivity analysis excluding insurance status was also performed and did not change the significance of any findings (eTable in the Supplement).

**Table 4.** Multivariable Regression Analysis of Participants With Diabetic Retinopathy as Outcome

Variable	Odds ratio (95% CI)	SE	*z* Score	*P* value
**Type 1 diabetes (n = 1169)**				
Duration of diabetes	1.18 (1.10-1.26)	0.04	4.51	<.001
Race and ethnicity				
Black or African American	1.79 (0.83-3.89)	0.71	1.49	.14
Hispanic	0.78 (0.31-1.92)	0.36	−0.55	.58
White	1 [Reference]	NA	NA	NA
Other^a^	1 (NA)	NA	NA	NA
Insurance				
Private/commercial	1 [Reference]	NA	NA	NA
Public/Medicaid	1.15 (0.58-2.30)	0.41	0.40	.69
Mean HbA_1c_ level	1.16 (0.99-1.36)	0.10	1.80	.07
Use of insulin pump	0.43 (0.20-0.93)	0.17	−2.16	.03
**Type 2 diabetes (n = 346)**				
Duration of diabetes	1.21 (1.02-1.45)	0.11	2.18	.03
Race and ethnicity				
Black or African American	1.08 (0.30-3.85)	0.70	0.11	.91
Hispanic^b^	1 (NA)	NA	NA	NA
White	1 [Reference]	NA	NA	NA
Other^a^^,^^b^	1 (NA)	NA	NA	NA
Insurance				
Private/commercial	1 [Reference]	NA	NA	NA
Public/Medicaid	1.95 (0.39-9.70)	1.60	0.82	.42
Mean HbA_1c_ level	0.96 (0.76-1.22)	0.12	−0.33	.74

Abbreviations: HbA_1c_, hemoglobin A_1c_; NA, not applicable.

^a^ The other category includes patients who were American Indian or Alaska Native (n = 1), Asian (n = 50), Native Hawaiian or Pacific Islander (n = 1), and did not specify race or ethnicity, specified other race or ethnicity, or for whom data on race or ethnicity were not available (n = 51). For this analysis, patients who were Hispanic or of other races and ethnicities were combined and compared with patients who were Black and White.

^b^ For the analysis of type 2 diabetes, Hispanic and other patients had either no observations in a particular category or too few to generate a result.

## Discussion

In this cross-sectional study, the overall prevalence of DR was 3.5%, which was consistent with that of other studies conducted in the post–Diabetes Control and Complications Trial era.^33,34,35^ A 2017 US-based study by Wang et al^15^ reported a higher prevalence of DR among youths with diabetes (14.4%) compared with our study. However, it is important to note the different study settings. The Wang et al^15^ study used insurance claims data and included participants receiving care from an optometrist or ophthalmologist, whereas our data were obtained from fundus photographic screening of participants in an endocrine clinic setting. Although patients with type 1 and type 2 diabetes had similar DR prevalence in our analysis, the Treatment Options for Type 2 Diabetes in Adolescents and Youth study of youths with types 2 diabetes found a higher prevalence (13.7%) of DR,^36^ which was likely owing to the higher mean age and longer duration of diabetes among participants in the Treatment Options for Type 2 Diabetes in Adolescents and Youth study. The SEARCH for Diabetes in Youth study also reported more complications of diabetes among youths with type 2 compared with type 1 diabetes and hypothesized that a lag time in the diagnosis of type 2 diabetes could have accounted for these differences.^35^ Their data were gathered from an outcomes visit occurring 5 to 15 years after initial participant selection, whereas our patients were selected as part of a DR screening program; thus, the participants in the present study were likely to be earlier in their disease course with a lower prevalence of DR.

An interesting finding of our analysis was that insulin pump use was associated with a lower prevalence of DR even after controlling for HbA_1c_ levels. Limited studies of this association have been reported given the difficulty in differentiating insulin pump use from multiple daily injections in analyses of intensive insulin therapy^7,37^; however, Downie et al^34^ have also previously documented this association in an Australian adolescent population. Bourry et al^38^ reported that pump use was a protective factor against progression of DR during pregnancy among women with prepregnancy DR. Our finding that insulin pump use was associated with lower rates of DR even when controlling for HbA_1c_ levels was unexpected. We postulate that this finding may be associated with a decrease in glycemic variability or an increase in time in range (ie, the percentage of time blood glucose levels remain within the 70-180 mg/dL range) among those receiving insulin pump therapy.^39^ Time in range and glycemic variability measures, such as coefficients of variation, have been increasingly cited as measures of glycemic control with clinical implications that capture variability missed by using HbA_1c_ as a measure^40^; however, these measures were not available in our study and would have required consistent use of a continuous glucose monitor by all participants.

In our study, participants who used insulin pumps had more frequent follow-up with their endocrinologists, which could suggest that these individuals received additional guidance in treatment optimization and had the resources, time, and motivation to work on improving their glycemic control, including optimizing their time in range. We also found that more patients with private vs public health insurance used insulin pumps. Although use of a continuous glucose monitor was not associated with a reduction in DR prevalence in our study, the increased use of closed-loop automated insulin delivery systems in the near future may likely further improve control and increase time in range, reducing DR prevalence.^41^

Several studies have noted racial disparities in DR, with Black or African American and Hispanic patients having a higher prevalence of DR.^35,42,43^ Thomas et al^43^ also documented a disparity in the use of diabetes technologies among non-White youths; however, to our knowledge, the association between pump use, race, and DR among youths has not been previously directly investigated. In our study, Black or African American patients were twice as likely to have DR compared with White patients. However, pump use, which was significantly associated with a lower prevalence of DR in our study, was significantly lower among Black or African American patients (27.0%) compared with White patients (59.1%; *P* < .001). After adjusting for pump use, duration of type 1 diabetes, insurance status, and HbA_1c_ level, the difference in DR prevalence by race was no longer significant. This result suggests that racial disparity in DR prevalence may be owing to factors associated with diabetes control and is consistent with the findings of previous studies.^19,35,43,44^ Given that patients who used insulin pumps in our study also had significantly fewer admissions for diabetic ketoacidosis than those who did not use pumps, the benefits of increased access to insulin pumps would likely be multifactorial.

Since collection of our data, the American Diabetes Association has updated its guidelines for eye examinations among youths with type 1 diabetes. They now recommend a dilated eye examination for those 11 years and older who have had diabetes for 3 to 5 years, and they included a statement suggesting that examinations could be spaced up to 4 years apart among patients perceived to be at lower risk.^20,32^ This change in recommendations is part of an increasing body of thought regarding the necessity of retinal imaging in younger children with type 1 diabetes.^45,46^ Even before the results of the Diabetes Control and Complications Trial were published, the Wisconsin Epidemiologic Study of Diabetic Retinopathy (WESDR) reported few cases of PDR in their population before age 20 years and no cases of PDR in patients younger than 15 years.^47^ Other studies have suggested that actionable DR in patients with type 1 diabetes is rare among those younger than 15 years and those receiving intensive insulin therapy with less than a 5-year duration of diabetes.^15,48,49,50^ Because other diabetes complications, such as nephropathy and neuropathy, commonly co-occur with DR,^51^ their presence should also be a factor that precipitates DR screening. Although we are likely to see a continued decrease in DR among young patients with type 1 diabetes because of the increased use of diabetes technologies (thus warranting less frequent screening), the opposite is likely to occur among those with type 2 diabetes, who may have DR at diagnosis and are at increased risk of developing DR at shorter diabetes durations.^52^ The only case of PDR in our study occurred in a girl aged 16 years who had type 2 diabetes for less than 2 years.

Notably, the 2021 American Diabetes Association guidelines specifically state that retinal photography is an acceptable modality for DR screening among youths with type 2 diabetes, but the guidelines do not mention retinal photography for those with type 1 diabetes.^20^ The use of fundus photography located in pediatric offices rather than ophthalmologic clinics in our study was likely helpful to our ability to form a large cohort of patients because increased ease of access to DR screenings increases adherence to guidelines.^26,53^

### Limitations

This study has limitations. These limitations include the differences in patient enrollment criteria between our 2 centers, with 1 center using prospective and the other using retrospective data collection. Therefore, although the patients at BCM/TCH were selected to meet the 2018 American Diabetes Association criteria for DR screening, some patients from JHU who did not meet guidelines for screening were included in our analysis. Although these patients comprised a smaller proportion of the total patients in the study, it is conceivable that this factor could have lowered our reported prevalence of DR.

In addition, the method for grading fundus images varied slightly between the 2 centers; JHU used 2 images per eye with grading by a retinal specialist, whereas BCM/TCH used 1 image per eye with initial grading by an optometrist, with positive images reviewed by a retinal specialist to confirm the diagnosis and grade of DR. Despite these differences, it has been previously reported that the grading of retinal images by different types of eye care professionals is unlikely to change the assessment of DR, especially in cases of clinically significant DR.^54^ The use of urban clinical centers allowed us to include a large and diverse population, but this approach does exclude patients who live in rural areas and do not have access to tertiary medical centers.

## Conclusions

This study found that the use of insulin pumps was associated with a lower likelihood of developing DR among patients with type 1 diabetes. Outcomes may improve further with the increased use of closed-loop or automated insulin delivery systems. Future studies could investigate whether improved access to diabetes care and technologies, such as insulin pumps, may mitigate the racial disparities in DR prevalence.

## Notes

Supplement. **eTable.** Multivariable Regression Analysis of Patients With Diabetic Retinopathy as Outcome Excluding Insurance
