# Supplementary material for: Clinical and Demographic Factors Associated With Diabetic Retinopathy Among Young Patients With Diabetes
Source: JAMA Netw Open. 2021 Sep 27;4(9):e2126126. doi: 10.1001/jamanetworkopen.2021.26126 (PMC8477260; doi:10.1001/jamanetworkopen.2021.26126)
Supplement: Supplement. — eTable. Multivariable Regression Analysis of Patients With Diabetic Retinopathy as Outcome Excluding Insurance [file jamanetwopen-e2126126-s001.pdf]

Supplementary Online Content

Ferm ML, DeSalvo DJ, Prichett LM, Sickler JK, Wolf RM, Channa R. Clinical and demographic factors associated with diabetic retinopathy among young patients with diabetes. *JAMA Netw Open*. 2021;4(9):e2126126. doi:10.1001/jamanetworkopen.2021.26126

eTable. Multivariable Regression Analysis of Patients With Diabetic Retinopathy as Outcome Excluding Insurance

This supplementary material has been provided by the authors to give readers additional information about their work.

© 2021 Ferm ML et al. *JAMA Network Open*.

eTable. Multivariable Regression Analysis of Patients With Diabetic Retinopathy as Outcome Excluding Insurance

**T1D (n=1207)**	Odds Ratio (95% Confidence Interval)	Standard Error	z	P>|z|
Duration of Diabetes	1.172 (1.093-1.256)	0.041	4.480	**<0.001**
Race (categorical)				
White (NH)	Reference			
Black/African American (NH)	1.843 (0.867-3.918)	0.709	1.590	0.11
Hispanic/Other	0.635 (0.266-1.517)	0.282	-1.020	0.31
Mean HbA1c	1.168 (0.996-1.369)	0.095	1.910	0.06
Pump (y)	0.440 (0.204-0.948)	0.172	-2.100	**0.04**

**T2D (n=376)**	Odds Ratio (95% Confidence Interval)	Standard Error	z	P>|z|
Duration of Diabetes	1.259 (1.076-1.474)	0.101	2.870	**0.004**
Race (categorical)				
White (NH)	Reference			
Black/African American (NH)	0.927 (0.274-3.138)	0.577	-0.120	0.90
Hispanic/Other	1.000	(omitted)		
Mean HbA1c	1.035 (0.837-1.280)	0.112	0.320	0.75

T1D = type 1 diabetes, T2D = type 2 diabetes, DR = diabetic retinopathy, NH = non-Hispanic, HbA1c = hemoglobin A1c

© 2021 Ferm ML et al. *JAMA Network Open*.
